# Microbial Response to Soil Liming of Damaged Ecosystems Revealed by Pyrosequencing and Phospholipid Fatty Acid Analyses

**DOI:** 10.1371/journal.pone.0168497

**Published:** 2017-01-04

**Authors:** Ramya Narendrula-Kotha, Kabwe K. Nkongolo

**Affiliations:** 1 Biomolecular Sciences Program, Laurentian University, Sudbury, Ontario, Canada; 2 Department of Biology, Laurentian University, Sudbury, Ontario, Canada; Universidade de Aveiro, PORTUGAL

## Abstract

**Aims:**

To assess the effects of dolomitic limestone applications on soil microbial communities’ dynamics and bacterial and fungal biomass, relative abundance, and diversity in metal reclaimed regions.

**Methods and Results:**

The study was conducted in reclaimed mining sites and metal uncontaminated areas. The limestone applications were performed over 35 years ago. Total microbial biomass was determined by Phospholipid fatty acids. Bacterial and fungal relative abundance and diversity were assessed using 454 pyrosequencing. There was a significant increase of total microbial biomass in limed sites (342 ng/g) compared to unlimed areas (149 ng/g). Chao1 estimates followed the same trend. But the total number of OTUs (Operational Taxonomic Units) in limed (463 OTUs) and unlimed (473 OTUs) soil samples for bacteria were similar. For fungi, OTUs were 96 and 81 for limed and unlimed soil samples, respectively. Likewise, Simpson and Shannon diversity indices revealed no significant differences between limed and unlimed sites. Bacterial and fungal groups specific to either limed or unlimed sites were identified. Five major bacterial phyla including *Actinobacteria*, *Acidobacteria*, *Chloroflexi*, *Firmicutes*, and *Proteobacteria* were found. The latter was the most prevalent phylum in all the samples with a relative abundance of 50%. *Bradyrhizobiaceae* family with 12 genera including the nitrogen fixing *Bradirhizobium* genus was more abundant in limed sites compared to unlimed areas. For fungi, *Ascomycota* was the most predominant phylum in unlimed soils (46%) while *Basidiomycota* phylum represented 86% of all fungi in the limed areas.

**Conclusion:**

Detailed analysis of the data revealed that although soil liming increases significantly the amount of microbial biomass, the level of species diversity remain statistically unchanged even though the microbial compositions of the damaged and restored sites are different.

**Significance and Impact of the study:**

Soil liming still have a significant beneficial effects on soil microbial abundance and composition > 35 years after dolomitic limestone applications.

## Introduction

Mining activities in the Greater Sudbury Region (GSR) in Northern Ontario (Canada) that started over century ago have resulted in serious damages to surrounding ecosystems. Logging and ore smelting led to a large scale of SO_2_ (sulphur dioxide) emissions and metal contamination (copper, Cu; iron, Fe; nickel, Ni and zinc, Zn) [1–5]. These have resulted in damaged ecosystems with reduced plant growth and population diversities within the region [2,3,5]. Remediation projects were initiated to restore the affected lands and to decrease industrial emissions. Building of a super chimney in 1972 reduced metal particulates and SO_2_ emissions by 50% and 85%, respectively [2]. To decrease acidity, 10 tons of limestones per hectare were applied in 1978. This liming was followed by land fertilization, grass and legume seeding [1,2]. In addition, since 1979, 12 million trees have been planted to complete the reclamation process [2,3,5].

Several studies investigating the effects of metal contamination on soil biology reported mixed results. Some found no changes in microbial biomass while others reported a decrease in microbial relative abundance associated with high levels of soil metal accumulation [4,6–8]. Extensive research has been conducted on the effects of liming on agricultural soils within the context of integrated soil management [2,9–13]. Overall, these studies showed that liming resulted in the mineralization of the soil solution due to its saturation with calcium (Ca) and in some cases with magnesium (Mg).

The addition of liming lowered soil acidity, reduced soil erosion and metal mobility, and resulted in an increase in organic matter [3,5,9]. Recent analysis revealed that soil liming in the GSR increased plant species richness and abundance and the overall plant ecosystem health [14–16]. This has led to improvement of soil fertility in limed sites compared to unlimed areas [14–16]. Relationship between soil pH and its effect on the activity and composition of microbial populations have been discussed in various reports [4,6,8–10,17–19]. Low pH inhibits the growth of soil bacteria in favor of more resistant fungi [18,20]. Liming of acidic soils creates improved environmental conditions for the development of acid-intolerant microbes, resulting in increased microbial biomass and soil respiration [2,13,21]. Also, soil type and management affect microbial and chemical responses once lime is applied [20]. Enhanced soil respiration as well as nitrogen mineralization have also been documented when lime is added to weakly acidic soils [10,22]. Pawlett *et al*. [11] reported that liming significantly altered PLFA profile (reduced bacterial fatty acids and did not affect fungal PLFA signature) and increased respiration rates. Other studies reported that application of fertilizers and lime resulted in the activation of biochemical processes; an increase in the relative abundance of prokaryotes, *Bacilli* and *Actinomycetes*; and a decrease in micromycetes [9,23]. In contrast, there are reports on the inhibitory effect of lime on some groups of prokaryotes such as actinomycetes that facilitate the mineralization of soil organic matter (SOM) [8,9].

Most of these studies focused on bacterial relative abundance in agricultural soil. We have a poor understanding of how liming affects microbial relative abundance, diversity and dynamic within a context of land reclamation of severely damaged ecosystems. Moreover, studies on the variation of fungi communities in response to soil liming are lacking. We hypothesize that liming increases bacterial and fungal biomass, relative abundance, diversity, and community composition.

The present report discusses for the first time the effect of soil liming on belowground diversity using PLFA and pyrosequencing analyses in the GSR. The main objectives of this study were to assess the long term effects of liming on 1) soil microbial biomass and composition (bacteria, fungi, and actinomycetes) and 2) bacterial and fungal population dynamic and diversity in reclaimed ecosystems.

## Materials and Methods

### Site characterization and sampling

The study was conducted in reclaimed mining sites in Northern Ontario, Canada (46°30′ N, 80°00′ W). The topography of the region is characterized by mosaic of rock outcrops, glacial till deposits, numerous lakes, and narrow valleys resulting from the Wisconsin glaciations [2]. The dominant soil type in the GSR area are podzols, which are well drained soils on sandy till with an organic top layer, a grey middle, and a reddish-brown lower layer [24]. Liming of this region was completed > 35 years ago through the Sudbury Regional Land Reclamation Program (Regreening Program) using dolomitic limestone [2,25].

Soil sampling was performed at four selected locations each with a reclaimed site (limed site) and its adjacent un-reclaimed or unlimed area. These targeted sites include Daisy Lake 2 (site 1), Wahnapitae Hydro-Dam (site 2), Kelly Lake (site 3), and Kingsway (site 4). At each of the four limed and four unlimed sites, 20 soil samples were collected in 2014 from the organic layer (0–5 cm in depth). Plant materials, stones, and residues were removed. Soil samples from each site were pooled and stored for a short period (in incubator at 30°C for soil chemical analyses; freezer for PLFA analysis for no more than 10 days before analysis). Microbial DNA was extracted from fresh samples within hours after soil sampling.

All the samplings took place on Laurentian University research fields and Crown lands. No specific permission was required to access the lands and collect samples.

### Soil chemistry analysis

Soil subsamples were sieved using a 2 mm mesh and air dried. Cation exchange capacity (CEC) was measured by using ammonium acetate extraction method at pH 7 [26]. CEC is an intrinsic property of soil defining the concentration of negatively charged sites on soil colloids that can adsorb exchangeable cations. The exchangeable cations that included aluminum, Al^3+^; calcium, Ca^2+^; iron, Fe^3+^; potassium, K^+^; magnesium, Mg^2+^; manganese, Mn^2+^; and sodium, Na^+^ were quantified by inductively coupled plasma mass spectrometry (ICP-MS) [14]. Soil pH was measured in de-ionized water and in neutral salt solution (0.1 M CaCl_2_) as described previously by Nkongolo *et al*. [14].

### Phospholipid fatty acid (PLFA) analysis

Soil samples (from four limed and four unlimed sites) were analyzed following the protocol described by Buyer and Sasser [27]. Mole percentage of each PLFA was used to determine bacterial and fungal biomass in soil. Total PLFA extracted from soil was used as an index of living microbial biomass [27]. The selected PLFAs for bacterial biomass include i15:0, a15:0, i16:0, 16:1ω9, 16:1ω7c, cy17:0, i17:0, a17:0, 18:1ω7 and cy19:0, while PLFA 18:2ω6 and 18:1ω9 were used for fungi. PLFAs such as 10Me, 20:3ω6/20:4ω6 and 12:0/16:1ω7/18:2ω9/18:2ω12/18:3ω9/18:3ω12/18:3ω15/polyunsaturated fatty acids were used for the identification of actinomycetes, protozoa, and eukaryotes, respectively.

### Microbial DNA extraction and purification

Microbial DNA was extracted from approximately 10 g of fresh soil (four limed and four unlimed) using the PowerMax^®^ soil DNA isolation kit for soil from MO BIO (cat # 12988–10) with the bead-beating protocol supplied by the manufacturer. The concentration of the DNA was determined using the flurochrome Hoechst 33258 (busdensimide) fluorescent DNA quantification kit from Bio-Rad (cat # 170–2480). Fluorescence intensity was measured using a BMG Labtech FLUOstar Optima microplate multi-detection reader in fluorescence detection mode. The quality was determined by running the samples on a 1% agarose gel. DNA samples were stored at -20°C until further analysis.

### PCR amplification and 454 pyrosequencing

Amplicon sequencing was performed at the MR DNA Molecular Research DNA laboratory (Shallowater, Texas, USA). Amplicon based analysis of the soil bacterial and fungal microbiota was assessed by high throughput sequencing of 16S rRNA gene and internal transcribed spacer (ITS) region. Tag-encoded FLX-titanium 16S rRNA gene amplicon pyrosequencing (bTEFAP) was performed using 16S universal Eubacterial primers 530F (5’ GTG CCA GCM GCN GCG G) and 1100R (5’ GGG TTN CGN TCG TTR) for amplifying the 600 bp region of 16S rRNA genes [28]. Fungal tag-encoded FLX amplicon pyrosequence (fTEFAP) was determined using ITS specific primers ITS1F (5’ TCC GTA GGT GAA CCT GCG G) and ITS4R (5’ TCC TCC GCT TAT TGA TAT GC) to amplify 600 bp fragment of the fungal ITS region [29]. The sequencing library was generated using a one-step PCR of 30 cycles using HotStarTaq Plus Master Mix Kit (Qiagen) under the following conditions: 94°C for 3 min followed by 28 cycles of 94°C for 30 sec, 53°C for 40 sec and 72°C for 1 minute, and a final elongation step at 72°C for 5 min [28]. Following PCR, all amplicon products from different samples were mixed in equal concentrations and purified using Agencourt Ampure beads. Tag-encoded FLX amplicon pyrosequencing analysis was performed using a Roche 454 FLX instrument with titanium reagents following the manufacturer’s guidelines with 3,000 sequence depth.

### Data processing

The sequencing data were processed using a proprietary analysis pipeline (MR DNA). Sequences were depleted of barcodes and primers. Short sequences (< 200 bp), sequences with ambiguous base calls, and sequences with homopolymer runs exceeding 6 bp were removed. Sequences were then denoised and chimeras were removed. OTUs (Operational Taxonomic Units) were defined following removal of singleton sequences, clustering at 3% divergence (97% similarity). OTUs were taxonomically classified using BLASTN (Basic Local Alignment Search Tool for Nucleotides) against a curated GreenGenes database (http://greengenes.lbl.gov; version 2011) and compiled into each taxonomic level.

### Statistical analyses

Data were analyzed using SPSS statistics version 20 for Windows. Association between soil pH and exchangeable cations were determined based on Pearson *r* correlation coefficients (p≤0.05). T-test was used to determine significant differences between limed and unlimed sites for CEC, microbial biomass (total PLFA), and composition (bacterial, actinomycetes and fungal PLFAs).

Chao1, Shannon index, Simpson index, and species richness and evenness for bacterial and fungal species diversity were analyzed using QIIME (version 1.8.0) [28,30–32]. Differences in frequency distribution for bacterial and fungal groups between limed and unlimed soils samples based on Kolmogorov-Smirnov test (non-parametric test) were determined using SPSS.

Pair wise comparisons (beta diversity) among the sites were performed using similarity indices (based on the presence/absence data), including Jaccard and Sorensen indices. Dissimilarity indices (based on abundance or relative abundance) including Bray-Curtis and Whittaker indices were calculated. Beta diversity was also estimated by computing weighed UniFrac (Qualitative) distances among sites. Weighed UniFrac variants are widely used in microbial ecology and they account for abundance of observed organisms. QIIME (version 1.8.0) was used to compute the within community diversity (alpha diversity), between community diversity (beta diversity), and UniFrac distances [30,33,34].

## Results

### Soil chemistry

**Fig 1:**
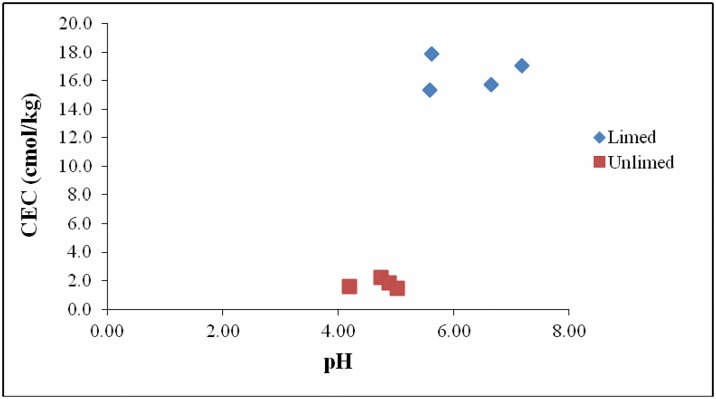
Patterns of pH and Cations Exchange Capacity (CEC) in samples from limed and unlimed sites in the Greater Sudbury Region (GSR) in Northern Ontario. Limed and Unlimed sites: Daisy Lake 2 (site 1), Wahnapitae Hydro-Dam (site 2), Kelly Lake (site 3), and Kingsway (site 4).

The pH in limed soils was significantly higher (pH 6.3) even > 35 years after dolomitic limestone applications (Table 1). Similar trend was observed for cation exchange capacity (CEC). Correlations between soil pH and CEC were positive (p≤0.05) for limed soil samples and negative (-0.64; p≤0.05) for samples from unlimed areas (Fig 1). As expected, the highest value of exchangeable Ca^2+^ and Mg^2+^ were recorded in limed soils (Table 1). Whereas, low values were obtained for exchangeable K^+^ and Na^+^ in both types of soils (Table 1). Total sum of exchangeable cation (Ca^2+^, Mg^2+^, K^+^ and Na^+^) values were 13.89 cmol/kg and 1.55 cmol/kg for limed and unlimed soils, respectively (Table 1).

**Table 1:** Mean values of basic cations (Ca^2+^, Mg^2+^, K^+^ and Na^+^), sum of cations, CEC and pH.

Characteristics	Limed sites	Unlimed sites
Ca^2+^ (cmol/kg)	10.52a ± 3.82	0.99b ± 0.48
Mg^2+^ (cmol/kg)	3.13a ± 1.36	0.26b ± 0.14
K^+^ (cmol/kg)	0.20a ± 0.04	0.25a ± 0.13
Na^+^ (cmol/kg)	0.04a ± 0.01	0.05a ± 0.03
Sum of Cations (cmol/kg)	13.89	1.55
CEC (cmol/kg)	16.30a ± 5.50	1.80b ± 0.90
pH (H_2_0)	6.30a ± 0.40	4.70b ± 0.20
pH (0.1M CaCl_2_)	5.80a ± 0.40	4.30b ± 0.30

Results are expressed as mean values ± standard error. Means in rows with a common letter are not significantly different based on T-test (p≥0.05). CEC: cation exchange capacity. Limed and Unlimed sites: Daisy Lake 2 (site 1), Wahnapitae Hydro-Dam (site 2), Kelly Lake (site 3), and Kingsway (site 4).

### Phospholipid fatty acid analysis

PFLA revealed a significant high level (p≤0.05) of total microbial biomass in limed sites compared to samples from unlimed areas (Table 2). The relative abundance levels of gram negative and gram positive bacteria, arbuscular mycorrhizal (AM), and other fungi followed the same trend (Table 2). There were positive correlations (p≤0.05) between gram negative bacterial biomass and pH (r = 0.71) as well as between AM fungi and pH (r = 0.59). But negative correlations (p≤0.05) were observed between gram positive bacterial biomass and pH (r = -0.49) and between fungal biomass and pH (r = -0.22).

**Table 2:** Microorganisms identified using phospholipid fatty acid (PLFA) analysis in soil samples from the Greater Sudbury Region (GSR). Data in ng/g.

Sites	Total microbial biomass	AM Fungi	Other Fungi	Gram Negative	Gram Positive	Other Eukaryote	Anaerobe	Actinomycetes
Limed sites	342.15a ± 35.61	16.10a ± 4.63	37.18a ± 6.10	164.90a ± 21.87	78.10a ± 9.38	8.49a ± 0.54	3.83a ± 0.68	33.56a ± 4.17
Unlimed sites	148.98b ± 61.47	5.67b ± 2.11	13.45b ± 5.34	58.92b ± 28.82	42.57b ± 16.82	4.35b ± 1.54	2.39a ± 0.84	21.63a ± 6.65

Results are expressed as mean values ± standard error. Means in columns with a common letter are not significantly different based on T-test (p≥0.05). Limed and Unlimed sites: Daisy Lake 2 (site 1), Wahnapitae Hydro-Dam (site 2), Kelly Lake (site 3), and Kingsway (site 4).

The ratio of gram positive to gram negative bacterial biomass was lower in the limed soil samples compared to unlimed (Table 3). The opposite trend was observed for 18w to 19cyclo ratio (Table 3). The ratio between fungal and bacterial biomass was very low for the two soil types (Table 3).

**Table 3:** Phospholipid fatty acid (PLFA) ratios of main microbial groups in soil samples from the Greater Sudbury Region (GSR).

Sites	Fungi/ Bacteria	Predator/ Prey	Gram +/Gram -	Saturated/ Unsaturated	Mono/ Poly	16w/ 17 cyclo	18w/ 19 cyclo
Limed sites	0.23a ± 0.05	0.04a ± 0.01	0.47a ± 0.02	0.87a ± 0.07	4.06a ± 0.98	2.20a ± 0.30	1.97a ± 0.47
Unlimed sites	0.20a ± 0.03	0.05a ± 0.00	0.72b ± 0.17	1.46b ± 0.10	3.19b ± 0.36	2.29a ± 0.08	0.63b ± 0.11

Results are expressed as mean values ± standard error. Means in columns with a common letter are not significantly different based on T-test (p≥0.05). Limed and Unlimed sites: Daisy Lake 2 (site 1), Wahnapitae Hydro-Dam (site 2), Kelly Lake (site 3), and Kingsway (site 4).

Palmitic acid (16:0) was the most common fatty acid averaging 12.98% for all soil samples. Other detected fatty acids included a15:0 (3.00%), i15:0 (6.73%), 16:1ω7c (7.21%), 16:1ω3c (3.30%), 10Me16:0 (4.53%), c17:0ω7c (3.25%), 18:0ω7c (11.26%), 18:1ω9c (8.40%), 18:2ω6c (7.49%), and c19:0ω7c (7.88%). These fatty acids were present in all the sites and made up about 78% and 73% of total fatty acid content in the limed and unlimed soil samples, respectively. Overall, monounsaturated fatty acids were more prevalent in the targeted sites followed by saturated, branched, and cyclo chain fatty acids.

### Pyrosequencing analyses

Overall, 63,814 and 42,979 sequences were generated for bacteria and fungi, respectively. The number was reduced to 61,630 and 41,588 for bacterial 16S rRNA and fungal ITS after trimming, removing chimeras, and omitting sequences shorter than 200 bp. Relative abundances of bacterial and fungal groups were estimated at the phylum, family, class and genus levels. All the bacterial sequences were classified while fungal sequences were unclassified. In fact, for the *Ascomycota* phylum, 48% of the sequences were not classified and for the *Basidiomycota*, this portion was 0.02%. Relative abundances of bacterial and fungal species are illustrated in S1 and S2 Tables. The reads generated in this project have been deposited in the NCBI Short Read Archive database (Accession number: SRP071853).

#### Bacterial community composition and diversity analysis

**Fig 2:**
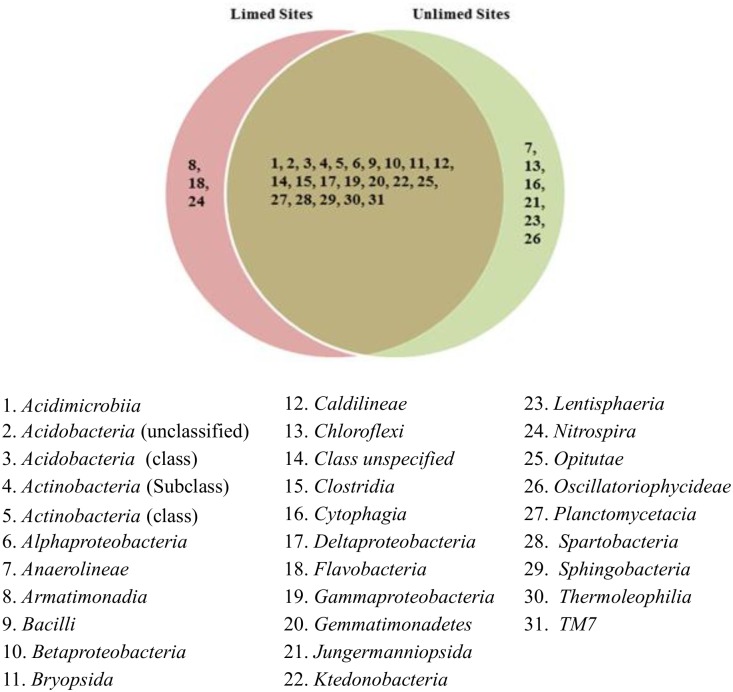
Venn diagram showing distribution of bacterial classes identified in limed and unlimed soil samples from the GSR. Limed and Unlimed sites: Daisy Lake 2 (site 1), Wahnapitae Hydro-Dam (site 2), Kelly Lake (site 3), and Kingsway (site 4).

**Fig 3:**
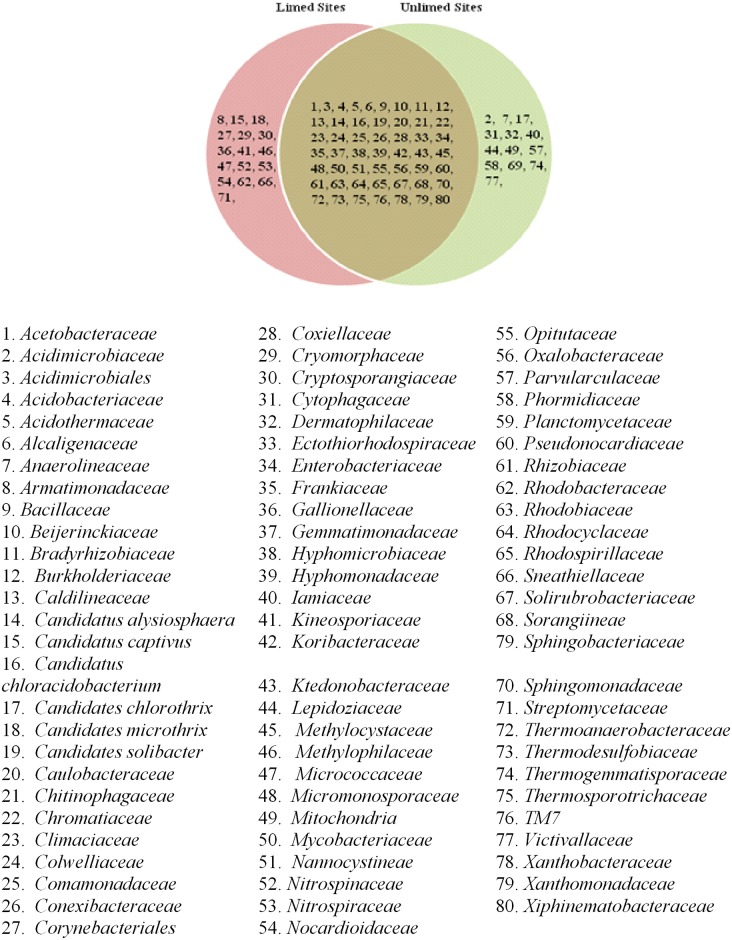
Venn diagram showing distribution of bacterial families identified in limed and unlimed soil samples from the GSR. Limed and Unlimed sites: Daisy Lake 2 (site 1), Wahnapitae Hydro-Dam (site 2), Kelly Lake (site 3), and Kingsway (site 4).

**Fig 4:**
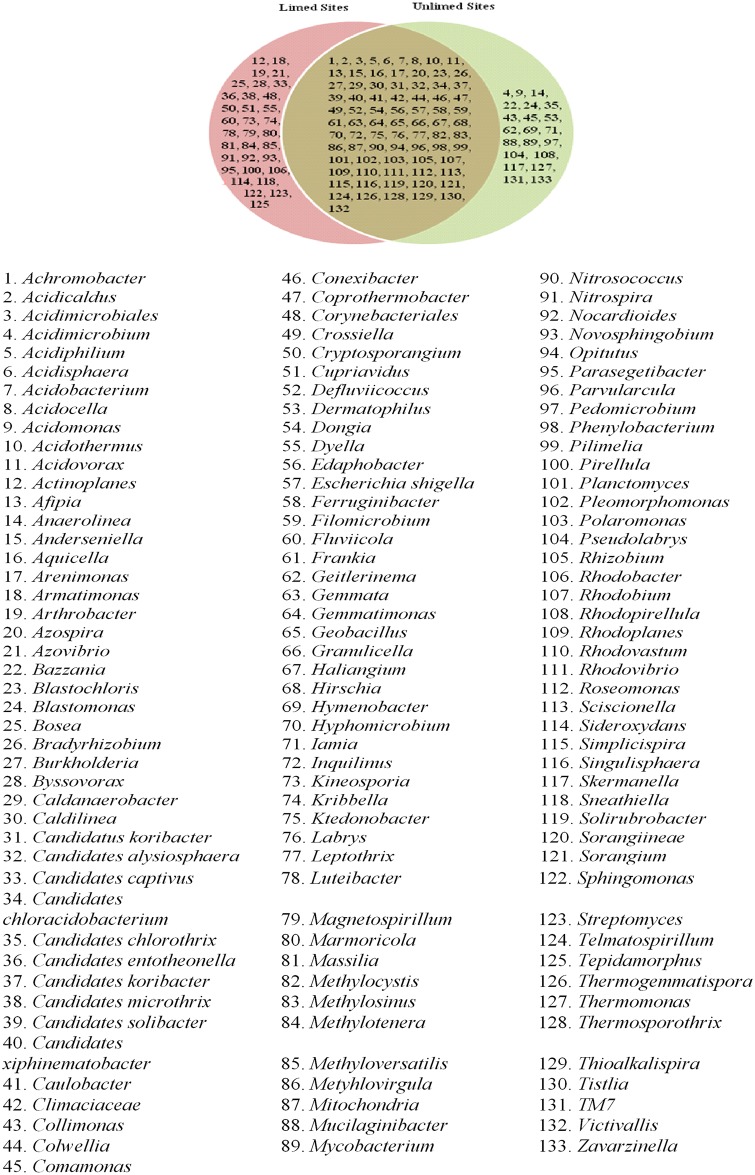
Venn diagram showing distribution of bacterial genera identified in limed and unlimed soil samples from the GSR. Limed and Unlimed sites: Daisy Lake 2 (site 1), Wahnapitae Hydro-Dam (site 2), Kelly Lake (site 3), and Kingsway (site 4).

Five major bacterial phyla were identified in the soil samples analyzed. They include *Actinobacteria*, *Acidobacteria*, *Chloroflexi*, *Firmicutes*, and *Proteobacteria*. They accounted for 96.01% of the phyla present in the soil samples. *Firmicutes* and *Chloroflexi* were the least abundant in all soil samples with a relative average abundance of 3.23% and 4.62%, respectively. They were mostly found in unlimed soils (Table 4). The most abundant phylum was *Proteobacteria* with a prevalence of 52.48%, followed by *Acidobacteria* (28.30%), and *Actinobacteria* (7.34%). *Acidobacteria* were more prevalent in unlimed soils compared to limed soils (p≤0.05) (Table 4). The most preponderant families in this phylum included *Bradyrhizobiaceae*, *Rhizobiaceae*, *Rhodobacteraceae*, *Rhodobiaceae*, *Rhodocyclaceae*, and *Rhodospirillaceae*. *Bradyrhizobiaceae* family with 12 genera including the nitrogen fixing *Bradirhizobium* genus was more abundant in limed sites compared to unlimed areas. In total, 31 bacterial classes, 80 families, and 133 genera were identified. Venn diagrams show different bacterial groups and their distribution (Figs 2–4).

**Table 4:** The main phylogenetic groups of bacteria and fungi, and their relative abundance in soil samples from the Greater Sudbury Region (GSR).

Phylum	Limed sites	Unlimed sites
**Bacteria**		
*Actinobacteria*	10.90a ± 3.81	3.77b ± 1.11
*Acidobacteria*	18.53a ± 6.17	38.15b ± 3.34
*Chloroflexi*	1.17a ± 0.67	8.06a ± 5.12
*Firmicutes*	1.88a ± 1.39	4.59a ± 2.24
*Proteobacteria*	64.74a ± 3.88	40.22b ± 5.45
**Fungi**		
*Ascomycota*	13.79a ± 3.92	46.00b ± 14.52
*Basidiomycota*	85.74a ± 4.07	49.20b ± 12.03
*Zygomycota*	0.46a ± 0.20	4.80b ± 1.92

Results are expressed as mean percentages ± standard error. Means in rows with a common letter are not significantly different based on T-test (p≥0.05). Limed and Unlimed sites: Daisy Lake 2 (site 1), Wahnapitae Hydro-Dam (site 2), Kelly Lake (site 3), and Kingsway (site 4).

No differences in the frequency distribution for bacterial taxonomies (family: p = 0.74; class: p = 0.29 and genera: p = 0.80) were observed between limed and unlimed soil samples based on Kolmogorov-Smirnov test (non-parametric test). At the genus level, *Solirubrobacter* and *Nocardioides* belonging to the *Actinobacterial* phylum were more abundant in limed sites compared to unlimed soil samples. Interestingly, *Nitrospira* class was found only in limed site whereas *Anaerolineaea*, *Chloroflexi*, *Jungermanniopsida*, *lentisphaeria*, *Oscillatoriophycideae* classes were found only in unlimed areas. Likewise, there were 25% of genera specific to limed sites and 16% to unlimed (Fig 4).

In total, 149 bacterial groups were identified in all the targeted sites (S1 Table) of which 121 were found in limed sites and 108 were present in unlimed soil samples (S1 Table). Further analysis revealed that 20 bacterial groups were present only in limed sites and 21 in unlimed soils (S3 Table). *Afipia broomeae*, *Afipia felis*, *Arenimonas* spp., *Bradyrhizobium* spp., *Candidates alysiosphaera* spp., *Defluviicoccus* spp., *Ktedonobacter* spp., *Labrys* spp., *Pedomicrobium* spp., *Pilimelia* spp. and *Pseudolabrys* spp. were more abundant (p≤0.05) in limed sites compared to unlimed areas (S1 Table). On the other hand, *Acidobacterium* spp., *Aquicell* spp., *Caldanaerobacter thermoanaerobacter tengcongensis*, *Candidates koribacter* spp., *Conexibacter* spp., *Filomicrobium* spp., *Gemmatimonas* spp., *Geobacillus* spp., *Mycobacterium riyadhense*, *Planctomyces* spp., *Sciscionella* spp., *Skermanella* spp., *Sorangium* spp., *Thermosporothrix* spp., *Thioalkalispira* spp. and *TM7* were prevalent in unlimed areas compared to soils from limed sites (S1 Table).

The average estimated minimum number of species (OTUs), Chao1 index, Shannon’s index, Simpson index, and species richness and evenness for the two soil types (limed and unlimed) are described in Table 5. Final dataset yielded 619 OTUs of which 463 were observed in limed and 473 in unlimed sites (Table 5). Chao1 values were higher in limed compared to unlimed soil samples (Table 5). However, there was no significant difference for Simpson index, Shannon index, and species richness and evenness between microbial populations from limed and unlimed sites (p≥0.05) (Table 5). In the present study, Jaccard and Sorenson’s similarity indices were low (Table 6). Whereas, high values were recorded for Bray-Curtis and Whittaker dissimilarity indices (Table 6). Further, we compared the sites with one another using clustering based on the weighed UniFrac (incorporates relative abundance) distances. Low Bray-Curtis and Whittaker index values were observed when the two types of sites (limed vs unlimed) were compared suggesting that the microbial communities are closely related to each other (S4 Table).

**Table 5:** Microbial diversity index values for bacterial and fungal communities from the Greater Sudbury Region (GSR).

	Chao 1	# of OTUs	Simpson Index	Shannon Index (H’)	Species Evenness	Species Richness
**Bacteria species**						
Limed sites	257a ± 24.93	463	0.86a ± 0.08	5.68a ± 0.22	0.52a ± 0.02	121
Unlimed sites	153b ± 39.38	473	0.78a ± 0.04	4.29a ± 0.87	0.51a ± 0.01	108
**Fungi species**						
Limed sites	37a ± 6.54	96	0.81a ± 0.12	2.59a ± 0.30	0.51a ± 0.01	59
Unlimed sites	27b ± 9.07	81	0.78a ± 0.03	2.32a ± 0.51	0.54a ± 0.03	51

Results are expressed as mean values ± standard error. Means in column with a common letter are not significantly different within bacteria and fungi species based on T-test (p≥0.05). Limed and Unlimed sites: Daisy Lake 2 (site 1), Wahnapitae Hydro-Dam (site 2), Kelly Lake (site 3), and Kingsway (site 4).

**Table 6:** Pair wise microbial diversity analysis for bacteria and fungi communities in the Greater Sudbury Region (GSR).

Sites	Jaccard’s Index (β_j_)	Sorenson’s Index (β_sor_)	Bray-Curtis Index (β_b_)	Whittaker Index (β_w_)
**Bacteria species**				
Limed and Unlimed sites	0.26	0.41	0.53	0.59
**Fungi species**				
Limed and Unlimed sites	0.27	0.42	0.81	0.58

Limed and Unlimed sites: Daisy Lake 2 (site 1), Wahnapitae Hydro-Dam (site 2), Kelly Lake (site 3), and Kingsway (site 4).

#### Fungal community composition and diversity analysis

Pyrosequencing analysis identified three phyla of fungi that included *Ascomycota*, *Basidiomycota*, and *Zygomycota* (Table 4). *Dibaeis* (lichen-forming fungi), *baeomyces* (lichenized fungi), *Dermateaceae* sp., (plant pathogens and decay plant materials), *Fusarium oxysporum* (plant parasites), *Phialocephala fortini* (mycorrhizal fungi), and *Pezizales* sp. (saprophytes) were the most dominant groups of *Ascomycota*. The majority of sequences in the *Basidiomycota* phylum matched ectomycorrhizal (EM fungi) and wood rotting fungi.

**Fig 5:**
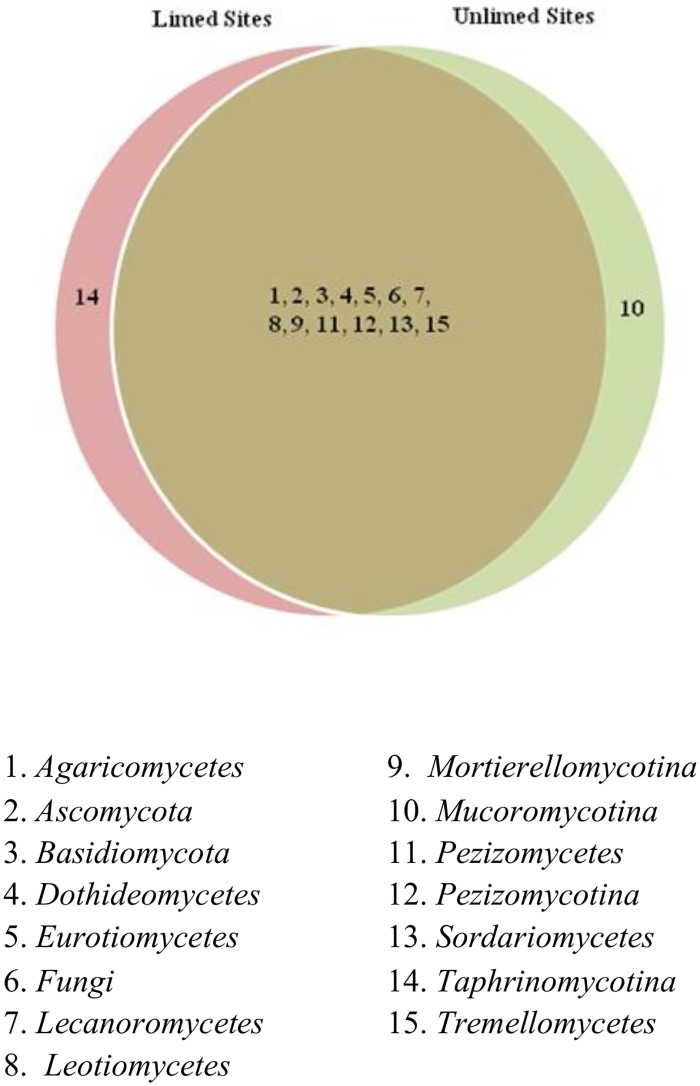
Venn diagram showing distribution of fungal classes identified in limed and unlimed soil samples from the GSR. Limed and Unlimed sites: Daisy Lake 2 (site 1), Wahnapitae Hydro-Dam (site 2), Kelly Lake (site 3), and Kingsway (site 4).

**Fig 6:**
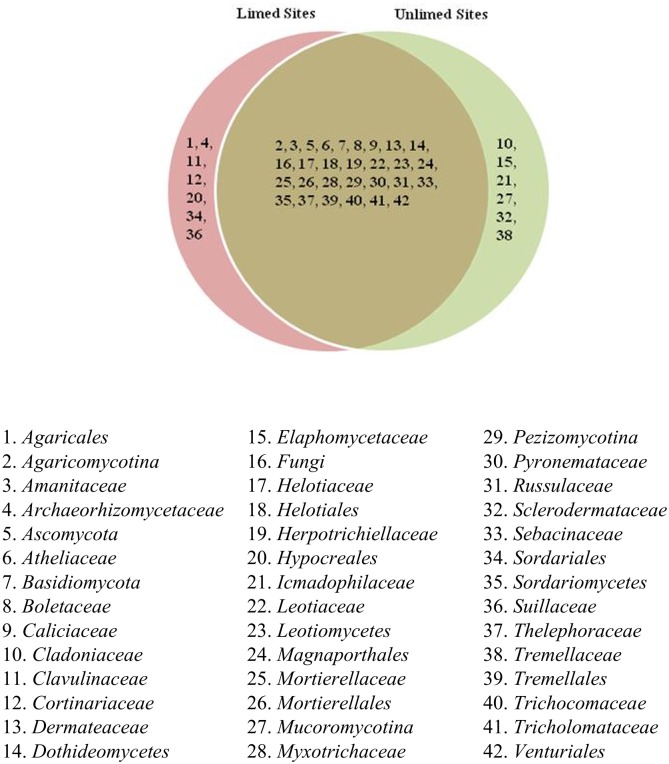
Venn diagram showing distribution of fungal families identified in limed and unlimed soil samples from the GSR. Limed and Unlimed sites: Daisy Lake 2 (site 1), Wahnapitae Hydro-Dam (site 2), Kelly Lake (site 3), and Kingsway (site 4).

**Fig 7:**
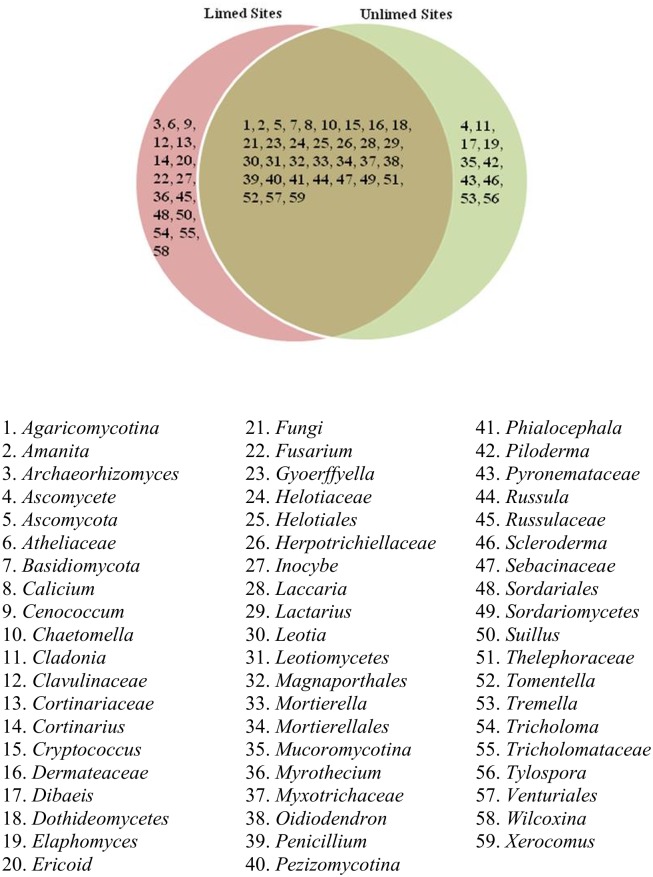
Venn diagram showing distribution of fungal genera identified in limed and unlimed soil samples from the GSR. Limed and Unlimed sites: Daisy Lake 2 (site 1), Wahnapitae Hydro-Dam (site 2), Kelly Lake (site 3), and Kingsway (site 4).

Overall, 15 fungal classes, 42 families, and 59 genera were identified. Venn diagrams show different fungal groups and their distribution (Figs 5–7). No differences in the frequency distribution for fungal groups were observed between limed and unlimed soils samples based on Kolmogorov-Smirnov test (non-parametric test) (family, p = 0.84; class, p = 0.77; and genera, p = 0.71). In total, 70 fungal species belonging to 59 genera were identified in limed and unlimed sites. They are described in S2 Table. In addition, 15 site-specific species were observed of which 10 were present only in limed and 5 in unlimed soil samples (S5 Table).

Several fungal species were more abundant in limed soils compared to unlimed sites. They include *Amanita muscaria*, *Cryptococcus podzolicus*, *Penicillium montanense*, *Pezizomycotina* sp., *Russula gracilis* and *Thelephoraceae* sp. (S2 Table). On the other hand, *Ascomycot*a sp., *Calicium salicinum*, *Dermateaceae* sp., *Dothideomycetes* sp., *Helotiaceae* sp., *Helotiales* sp., *Herpotrichiellaceae* sp., *Laccaria proxima*, *Leotia viscose*, *Mortierellales* sp., *Oidiodendron maius*, *Russula* sp., *Russula sphagnophila* and *Sebacinaceae* sp. were prevalent in unlimed areas compared to limed sites (p≤0.05) (S2 Table). In addition, several fungal genera known for their roles as human, plant and animal pathogens were found. They include *Dothideomycetes*, *Fusarium*, *Herpotrichiellaceae*, *Leotiomycetes*, *Magnaporthales*, *Mortierella*, *Pezizales*, *Scleroderma* and *Sordariomycetes*. Overall, 27% of genera were specific to limed sites and 17% to unlimed (Fig 7).

A total of 119 OTUs were identified of which 96 were found in limed and 81 in unlimed sites (Table 5). Estimated Chao1 values revealed significant differences between limed and unlimed sites (p≤0.05) (Table 5). But no significant differences between limed and unlimed sites were observed based on Simpson index, Shannon index, and species evenness data (p≥0.05) (Table 5). The study showed no change in species richness among limed and unlimed soil samples (Table 5). Further analysis indicated a weak negative correlation between all the fungal diversity parameters and pH. Pair wise comparisons among soil samples revealed low similarity indices or high dissimilarity indices (Table 6). Distance matrix values were high among samples (S6 Table).

## Discussion

The pH in limed soils was significantly higher than in unlimed areas reflecting the addition of dolomitic limestone to soils > 35 years ago. These results are consistent with data reported in other studies [4,6,14,16]. Likewise, strong positive correlations between soil pH and CEC reflect the data documented in other soil analysis under different conditions [15,35,36]. PLFA and pyrosequencing data demonstrate that microbial biomass and relative abundance were significantly higher in limed sites compared to the adjacent unlimed areas. Although several bacterial and fungal groups were present in all the sites, 25% of bacterial genera were specific to limed sites and 16% to unlimed areas. Similarly, the proportions of fungal genera specific to limed and unlimed sites were 27% and 17%, respectively. Chao1 values were higher for limed sites compared to unlimed areas for both bacteria and fungi. But the number of OTUs, and the levels of Simpson and Shannon diversity indices, and species evenness were not affected by liming.

### Phospholipid fatty acid analysis

PLFA analysis was used to determine soil microbial responses to liming. The results revealed an increase in total microbial biomass, AM fungi, other fungi, other eukaryote, actinomycetes, gram positive, and gram negative bacteria in limed sites compared to unlimed soil samples. This is likely the result of soil pH on microbial community [3,5,20,37]. Soils treated with different types of limes have also been found to change PLFA composition [9,37].

The relative proportion of branched PLFAs (17:0 and 18:0) and iso- and anteiso branched PLFAs (i15:0, i16:0, i16:1, and i17:0) were higher in limed soils compared to samples from unlimed areas suggesting that liming stimulated gram positive bacterial populations. Similar trend was observed for gram negative bacteria which were more abundant in limed soils compared to unlimed soil samples. PLFAs that are used as indicators of gram negative bacteria include 16:1ω5, 16:1ω9, 17:1ω9, cy17:0, 18:1ω7 and cy19:0 [37,38]. Overall, a predominance of gram negative over gram positive bacteria was observed in the two types of soils. This is usually common under stress conditions [5,37,38].

It has been demonstrated that acidic soil conditions enhance the development and activity of fungi relative to those of bacteria [5,18,20,37,39]. In the present study, 16:0 and 18:1ω9 PLFA considered as reliable indicators of fungal biomass were higher concentrations in unlimed soils compared to limed sites. But the concentration of PLFA 18:2ω6 another indicator of fungal biomass was lower in unlimed areas compared to limed sites. PLFA 18:1ω9 and 18:2ω6 are not exclusive to fungi since they are present in many eukaryotic organisms, including plants [5,40]. Therefore, some of these PLFAs identified in fungi especially in limed sites, might be from plant materials. In general, 16:1ω5 and 18:1ω7 are good indictors of AM fungi [41,42]. Both PLFAs were slightly more abundant in limed sites compared to unlimed samples. Overall, fungal biomass was significantly higher in limed compared to unlimed soil samples. In addition to the biomass and community structure determinations, PLFA analysis has been used to get an insight understanding of the physiological status of the microbes. The increase in trans/cis, saturated/unsaturated, and cy17:0/16:1ω7c ratios in the unlimed sites are consistent with stressed environmental conditions [38]. Low ratio of trans to cis-monoenoic unsaturated fatty acids, fungi to bacteria, and a high proportion of cyclopropyl fatty and gram negative PLFAs in all soil samples confirm that the region is still under environmental stress.

### Pyrosequencing analysis

Pyrosequencing has been used to analyze bacteria population dynamics [31–33]. In the present study, analysis of two types of soil samples showed a higher relative abundance of bacterial species and genera compared to fungi. In total, 149 bacterial groups were identified belonging to 133 genera. For fungi, 70 fungal groups corresponding to 59 genera were identified in the targeted sites.

Bacterial communities are directly influenced by soil pH as most bacterial taxa exhibit narrow growth tolerances [7,15,18,43]. Rousk *et al*. [18] and Ferandez-Calvino and Baath [43] reported that deviations of 1.5 pH units can reduce bacterial community’s activity by 50%. The shifts in the relative abundances of specific taxonomic groups across pH gradient are similar to the pH responses observed in other studies [18,43]. For instance, the relative abundance of *Acidobacteria* has been shown to increase when soil pH decreases [13,18,44]. Rousk *et al*. [18] and Lauber *et al*. [33] reported a strong positive correlation between relative abundance of *Bacteriodetes* and pH. But the results of the present study showed an opposite trend. The increase of *Bacteriodetes* in unlimed sites could be related to site characteristics such as carbon availability or soil moisture. The high relative abundance of *Proteobacteria* in limed sites is in agreement with Rousk *et al*. [18] and Lauber *et al*. [33] who reported an increase of these bacteria associated with a higher availability of carbon and high pH. *Proteobacteria* are members of photosynthetic and nitrogen fixing bacteria. The bacterial communities of those functional species play an important role in carbon, nitrogen cycles and in maintaining integrity of the ecosystem [33]. In fact, the targeted limed sites showed a high level of forest complexity and diversity compared to unlimed areas [16].

Bacterial community composition was also examined at the genus level. *Acidobacterium*, *Afipia*, *Aquicella*, *Bradyrhizobium*, *Geobacillus*, *Granulicella*, *Nitrosococcus*, *Rhodoplanes*, *Skermanella*, *Solirubrobacter*, *Thermosporothrix*, and *Thioalkalispira* were the most predominant genera in all the sites. The composition and distribution of these 12 common bacterial genera varied between limed and unlimed soil samples. Photosynthetic bacteria, such as *Rhodobacter* was found only in limed soils. These bacteria are known to play an irreplaceable role in carbon cycling and material transformation, and their diversities affect the process of nitrogen cycle [31]. *Rhizobium* present in all soil samples is a major contributor to the global nitrogen cycle as it forms a symbiotic nitrogen fixation with many plants. It also improves soils fertility, promotes circulation of soil materials and increases soil microbial activity [31]. The relative abundance of *Buckholderia* observed in the unlimed soil is consistent with the recent classification of this genus as an acid tolerant group [45]. *Bradyrhizobia* are often found in acid soils and they were identified in both limed and unlimed soil samples. In addition, several acid tolerant bacterial species were identified in both soil types.

Bacterial groups that grow in stressed environment and are involved in nitrogen fixation, carbon cycling and iron oxidation, were found in targeted sites at various levels of relative abundance. They are usually present in sludge and waste water treatments. *Nitrospira* bacteria that were observed only in limed sites in the present study are part of a nitrification process which is important in the biogeochemical nitrogen cycle. Overall, a higher percentage of gram negative bacterial species compared to gram positive were identified in all the sites. This is in agreement with PLFA analysis data.

#### Fungal populations

While many reports have limited their pyrosequencing analysis only on bacterial communities this study assessed fungal populations in all the sites as well. Recent studies using pyrosequencing technique have revealed that relative abundance of fungi is not affected by pH and that fungal diversity is only weakly associated to pH [18,21,46]. We confirmed that the composition of fungal community is weakly related to soil pH. This demonstrates that fungi exhibit wider pH ranges for optimal growth. The optimum level of pH varies from 5 to 9 [4,6,21,39]. Overall, pyrosequencing data are consistent with PLFA results revealing a much lower fungal biomass compared to bacteria in limed and unlimed sites.

The relative abundance of *Basidiomycota* was higher in limed soils compared to samples from unlimed sites. An opposite trend was observed for *Ascomycota*. The main *Ascomycota* detected in this study were classified as lichen forming fungi (*Calicium salicinum*, *Cladonia coniocraea*, *Dibaeis baeomyces*), little/wood decomposers (*Dermateaceae* sp., *Sordariomycetes* sp., *Tremella diploschistina*), plant parasites (*Dothideomycetes* sp., *Fusarium oxysporum*), human pathogens (*Herpotrichiellaceae* sp.), endophytes (*Gyoerffyella* sp., *Phialocephala fortinii*) and saprotrophs (*Venturiales* sp.). *Basidiomycetes* included EM fungi and decay organisms of plant residues. EM fungi colonize plant roots and help plants to access nutrients such as phosphorous from soil [32,47]. Several decomposers involved in the decomposition of hard woody organic matters and in the conversion of organic matters into fungal biomass, carbon dioxide (CO_2_) and organic acids were also present in the targeted sites. Compared to *Ascomycota* and *Basidiomycota*, a low relative abundance of *Zygomycota* was observed in all the soil samples analyzed. *Mortierella sp*. and *Mortierellales* sp. from the phylum *Zygomycota* were more abundant in unlimed compared to limed samples. These species mineralize readily dissolved organic substrates rather than breaking down soil litter polymers [48]. Members of the *Taphrinomycotina* were found only in limed sites. In general they range from simple and yeast-like to filamentous. Their role in the environment is not well documented.

#### Bacterial and fungal diversity

Estimates of diversity based on DNA sequencing are more accurate than culture techniques. In this study, relative abundance and diversity were estimated by the number of OTUs, Chao1, Shannon index and Simpson index. OTU is a widely used construct of clustered sequence data where each OTU represents a different microbial (bacterial or fungal) population in the community. These approaches are providing estimates of diversity at > 10,000 species (OTUs) of bacteria per gram of soil [49]. Dimitriu and Grayston [13] looked at bacterial diversity across reclaimed and natural boreal forest in British Columbia and reported 577 OTUs for bacteria. This is consistent with the results of the present study on the damaged and reclaimed sites in the GSR. Chao1 estimates species richness from the rarefaction of observed sequences present in a community. This index is based on the number of rare classes (i.e. OTUs or species) in a sample [34]. Chao1 estimates for bacterial species richness in soils were higher compared to fungi. Importantly, Chao1 estimates were higher in limed sites compared to unlimed areas for both bacteria and fungi. But this study revealed that liming has no effects on overall bacterial and fungal diversity based on Shannon and Simpson indices. The level of diversity in fungal communities was lower than bacterial diversity based on Shannon diversity index.

For beta measures, Jaccard and Sorenson indices are based on the presence/absence data [30,50]. Low values for Jaccard and Sorenson indices, for both bacteria and fungi indicate high dissimilarity (high beta diversity) between limed and unlimed areas. High values for Bray-Curtis and Whittaker dissimilarity indices indicate that there is a high number of species that are different between the two types of sites (high beta diversity). Overall, both similarity and dissimilarity indices reveal significant difference between limed and unlimed sites.

In addition, weighed UniFrac distances which take into account differences in relative abundance of taxa between limed and unlimed sites showed that for bacterial communities, limed sites are closely related to each other. Whereas for fungal communities, high distance values indicated that the sites are different from each other.

In the present study, both alpha and beta diversity indices were used. Our data show low levels of diversity within each group of sites (limed and unlimed) and low similarity among the two types of sites. This indicates that although liming increases significantly the amount of microbial biomass, the level of species diversity in limed compared to unlimed areas remain unchanged (and low) even though the microbial compositions are not similar. Ecological and soil properties such as vegetation type, soil texture, soil type, moisture and nutrient concentrations could play a significant role in microbial community composition and relative abundance.

### Pyrosequencing and PFLA comparison

In general, PLFA-based methods are rapid, inexpensive, sensitive and reproducible. There are a number of scientific publications based on PFLA analysis that have increased our understanding of the soil ecosystem. Unlike pyrosequencing, PLFA has limitations such as overlap in the composition of microorganisms and the specificity of PLFAs signature. For the present study, PFLA data revealed significant difference in microbial biomass among the limed and unlimed sites. But these differences were not always detected by pyrosequencing. In this study, the combination of PLFA and pyrosequencing analyses provided a more complete assessment of microbial relative abundance, structure, and diversity.

## Conclusions

In the present study, soil microorganisms were studied to assess the effect of liming on bacterial and fungal communities. This study is novel as it describes the effect of liming on microbial relative abundance and diversity > 35 years after dolomitic limestone applications. The combination of both PFLA and pyrosequencing validate our findings. Soil analysis revealed an increase in soil pH (6.3) and CEC (16.30 cmol/kg) in limed sites compared to unlimed areas. Total microbial biomass, bacterial and fungal relative abundance were higher in limed sites compared to unlimed samples. Chao 1 estimates followed the same trend. But the total number of OTUs in limed and unlimed soil samples for bacteria and fungi were similar. Likewise, Simpson and Shannon diversity indices revealed no significant differences between limed and unlimed sites. Bacterial and fungal group’s specific to limed and unlimed sites were identified. Overall, the results of the present study show that soil liming increase the amount of microbial biomass but have limited effects on bacterial and fungal diversities > 35 years after dolomitic applications. Further studies will target in details the effects of organic matter content and metal contamination on microbial relative abundance and diversity.

## Supporting Information

S1 Table Bacterial groups identified in soil samples from the Greater Sudbury Region (GSR) and their relative abundance. (DOCX)

S2 Table Fungal groups identified in soil samples from the Greater Sudbury Region (GSR) and their relative abundance. (DOCX)

S3 Table Site—specific bacterial groups identified in the Greater Sudbury Region (GSR) and their relative abundance. (DOCX)

S4 Table Weighed UniFrac distance matrix between sites for bacterial community. (DOCX)

S5 Table Site—specific fungal groups identified in the Greater Sudbury Region (GSR) and their relative abundance. (DOCX)

S6 Table Weighed UniFrac distance matrix between sites for fungal community. (DOCX)

## References

[1] Winterhaider K. The use of manual surface seeding, liming & fertilization in the reclamation of acid metal-contaminated land in the Sudbury, Ontario mining and smelting region of Canada. Environ Technol Lett. 1983;4: 209–216.

[2] Winterhalder K. Environmental degradation and rehabilitation of the landscape around Sudbury, a major mining and smelting area. Environ Rev. NRC Research Press Ottawa, Canada; 1996;4: 185–224. Available: http://www.nrcresearchpress.com/doi/abs/10.1139/a96-011

[3] Narendrula R, Nkongolo KK, Beckett P. Comparative soil metal analyses in Sudbury (Ontario, Canada) and Lubumbashi (Katanga, DR-Congo). Bull Environ Contam Toxicol. 2012;88: 187–192. 10.1007/s00128-011-0485-7 22139330

[4] Goupil K, Nkongolo K. Assessing soil respiration as an indicator of soil microbial activity in reclaimed metal contaminted lands. Am J Environ Sci. Science Publications; 2014;10: 403–411. Available: http://thescipub.com/abstract/10.3844/ajessp.2014.403.411

[5] Narendrula R, Nkongolo KK. Fatty Acids Profile of Microbial Populations in a Mining Reclaimed Region Contaminated with Metals: Relation with Ecological Characteristics and Soil Respiration. J Bioremediation Biodegrad. 2015;6: 1–9.

[6] Goupil K, Nkongolo KK, Nasserulla S. Characterization of fungal communities in limed and unlimed lands contaminated with metals: phospholipid fatty acid (PLFA) analysis and soil respiration. Am J Biochem Biotechnol. Science Publications; 2015;11: 45–56. Available: http://thescipub.com/abstract/10.3844/ajbbsp.2015.45.56

[7] Anand M, Ma K-M, Okonski A, Levin S, McCreath D. Characterising biocomplexity and soil microbial dynamics along a smelter-damaged landscape gradient. Sci Total Environ. 2003;311: 247–259. 10.1016/S0048-9697(03)00058-5 12826396

[8] Wang Y, Shi J, Wang H, Lin Q, Chen X, Chen Y. The influence of soil heavy metals pollution on soil microbial biomass, enzyme activity, and community composition near a copper smelter. Ecotoxicol Environ Saf. 2007;67: 75–81. 10.1016/j.ecoenv.2006.03.007 16828162

[9] Bakina LG, Chugunova M V, Zaitseva TB, Nebol’sina ZP. The effect of liming on the complex of soil microorganisms and the humus status of a soddy-podzolic soil in a long-term experiment. Eurasian Soil Sci. 2014;47: 110–118.

[10] Fuentes JP, Bezdicek DF, Flury M, Albrecht S, Smith JL. Microbial activity affected by lime in a long-term no-till soil. Soil Tillage Res. 2006;88: 123–131.

[11] Pawlett M, Hopkins DW, Moffett BF, Harris JA. The effect of earthworms and liming on soil microbial communities. Biol Fertil Soils. 2009;45: 361–369.

[12] Adeniyan ON, Ojo AO, Akinbode OA, Adediran JA. Comparative study of different organic manures and NPK fertilizer for improvement of soil chemical properties and dry matter yield of maize in two different soils. J Soil Sci Environ Manag. 2011;2: 9–13.

[13] Dimitriu PA, Grayston SJ. Relationship between soil properties and patterns of bacterial beta-diversity across reclaimed and natural boreal forest soils. Microb Ecol. 2010;59: 563–73. 10.1007/s00248-009-9590-0 19830478

[14] Nkongolo KK, Spiers G, Beckett P, Narendrula R, Theriault G, Tran A, et al Long-term effects of liming on soil chemistry in stable and eroded upland areas in a mining region. Water Air Soil Pollut. 2013;224: 1–14. Available: 10.1007/s11270-013-1618-x

[15] Nkongolo KK, Michael P, Theriault G, Narendrula R, Castilloux P, Kalubi KN, et al Assessing Biological Impacts of Land Reclamation in a Mining Region in Canada: Effects of Dolomitic Lime Applications on Forest Ecosystems and Microbial Phospholipid Fatty Acid Signatures. Water, Air, Soil Pollut. 2016;227: 1–13. Available: http://link.springer.com/10.1007/s11270-016-2803-5

[16] Theriault G, Nkongolo KK, Narendrula R, Beckett P. Molecular and ecological characterisation of plant populations from limed and metal-contaminated sites in Northern Ontario (Canada): ISSR analysis of white birch (Betula papyrifera) populations. Chem Ecol. 2013;29: 573–585. Available: http://www.tandfonline.com/doi/abs/10.1080/02757540.2013.820715

[17] Mühlbachová G, Tlustoš P. Effects of liming on the microbial biomass and its activities in soils long-term contaminated by toxic elements. Plant, Soil Environ. 2006;52: 345–352.

[18] Rousk J, Bååth E, Brookes PC, Lauber CL, Lozupone C, Caporaso JG, et al Soil bacterial and fungal communities across a pH gradient in an arable soil. ISME J. 2010;4: 1340–51. 10.1038/ismej.2010.58 20445636

[19] Tate RL. Soil microbiology. New York, USA: John Wiley; 2000.

[20] Pennanen T, Fritze H, Vanhala P, Kiikkilä O. Structure of a microbial community in soil after prolonged addition of low levels of simulated acid rain. 1998;64: 2173–2180. 10.1128/aem.64.6.2173-2180.1998 PMC106295 9603831

[21] Beales N. Adaptation of microorganisms to cold temperatures, weak acid preservatives, low pH, and osmotic stress: A review. Compr Rev Food Sci Food Saf. 2004;3: 1–20. 10.1111/j.1541-4337.2004.tb00057.x 33430556

[22] Curtin D, Campbell CA, Jalil A. Effects of acidity on mineralization: pH-dependence of organic matter mineralization in weakly acidic soils. Soil Biol Biochem. 1998;30: 57–64.

[23] Kamal S, Prasad R, Varma A. Soil Microbial Diversity in Relation to Heavy Metals In: Sherameti I, Varma A, editors. Soil Heavy Metals. New York, USA: Springer Berlin Heidelberg; 2010 pp. 31–63. 10.1007/978-3-642-02436-8_3

[24] Brown K. Hydrological response of Junction Creek, Sudbury Ontario, to rainfall events, fall 2005. Laurentian University 2006.

[25] Lautenbach WE, Miller J, Beckett PJ, Negusanti JJ, Winterhalder K. Municipal Land Restoration Program: The Regreening Process In: Gunn J, editor. Restoration and recovery of an industiral region. New York, USA: Springer-Verlag; 1995 pp. 109–122.

[26] Lavkulich L. Methods manual Pedology Laboratory, Department of Soil Science Vancouver, British Colombia; 1981.

[27] Buyer JS, Sasser M. High throughput phospholipid fatty acid analysis of soils. Appl Soil Ecol. 2012;61: 127–130.

[28] Dowd SE, Sun Y, Wolcott RD, Domingo A, Carroll JA. Bacterial tag-encoded FLX amplicon pyrosequencing (bTEFAP) for microbiome studies: bacterial diversity in the ileum of newly weaned Salmonella-infected pigs. Foodborne Pathog Dis. 2008;5: 459–72. 10.1089/fpd.2008.0107 18713063

[29] Schoch CL, Seifert KA, Huhndorf S, Robert V, Spouge JL, Levesque CA, et al Nuclear ribosomal internal transcribed spacer (ITS) region as a universal DNA barcode marker for Fungi. Proc Natl Acad Sci U S A. 2012;109: 6241–6246. 10.1073/pnas.1117018109 22454494 PMC3341068

[30] Kuczynski J, Stombaugh J, Walters WA, González A, Caporaso JG, Knight R. Using QIIME to analyze 16S rRNA gene sequences from microbial communities. Curr Protoc Bioinformatics. NIH Public Access; 2011;Chapter 10: Unit 10.7. 10.1002/0471250953.bi1007s36 PMC3249058 22161565

[31] Li Y, Chen L, Wen H, Zhou T, Zhang T, Gao X. 454 pyrosequencing analysis of bacterial diversity revealed by a comparative study of soils from mining subsidence and reclamation areas. J Microbiol Biotechnol. 2014;24: 313–23. 24296455 10.4014/jmb.1309.09001

[32] Lim YW, Kim BK, Kim C, Jung HS, Kim B-S, Lee J-H, et al Assessment of soil fungal communities using pyrosequencing. J Microbiol. 2010;48: 284–289. 10.1007/s12275-010-9369-5 20571944

[33] Lauber CL, Hamady M, Knight R, Fierer N. Pyrosequencing-based assessment of soil pH as a predictor of soil bacterial community structure at the continental scale. Appl Environ Microbiol. 2009;75: 5111–5120. 10.1128/AEM.00335-09 19502440 PMC2725504

[34] Chao A. Nonparametric estimation of the number of classes in a population. Scand J Stat. 1984;11: 265–270.

[35] Tomasic M, Zgorelec Z, Jurisic A, Kisic I. Cation exchange capacity of dominant soil types in the republic of croatia. J Cent Eur Agric. 2013;14: 937–951.

[36] Moreira A, Fageria NK. Soil chemical attributes of Amazonas State, Brazil. Commun Soil Sci Plant Anal. 2009;40: 2912–2925.

[37] Pennanen T. Microbial communities in boreal coniferous forest humus exposed to heavy metals and changes in soil pH—a summary of the use of phospholipid fatty acids, Biolog^®^ and 3H-thymidine incorporation methods in field studies. Geoderma. 2001;100: 91–126.

[38] Kaur A, Chaudhary A, Kaur A, Choudhary R, Kaushik R. Phospholipid fatty acid—A bioindicator of environment monitoring and assessment in soil ecosystem. Curr Sci. 2005;89: 1103–1112.

[39] Smith JL, Doran JW. Measurement and use of pH and electrical conductivity for soil quality analysis In: Doran JW, Jones AJ, editors. Methods for Assessing Soil Quality, SSSA Spec Publ 49. Madison, Wisconsin, USA: Soil Science Society of America; 1996 pp. 169–185.

[40] Frostegard A, Baath E. The use of phospholipid fatty acid analysis to estimate bacterial and fungal biomass in soil. Biol Fertil Soils. 1996;22: 59–65.

[41] Wang FY, Hu JL, Lin XG, Qin SW, Wang JH. Arbuscular mycorrhizal fungal community structure and diversity in response to long-term fertilization: a field case from China. World J Microbiol Biotechnol. 2011;27: 67–74.

[42] Koranda M, Kaiser C, Fuchslueger L, Kitzler B, Sessitsch A, Zechmeister-Boltenstern S, et al Fungal and bacterial utilization of organic substrates depends on substrate complexity and N availability. FEMS Microbiol Ecol. 2014;87: 142–52. 10.1111/1574-6941.12214 24024589

[43] Fernández-Calviño D, Bååth E. Growth response of the bacterial community to pH in soils differing in pH. FEMS Microbiol Ecol. 2010;73: 149–56. 10.1111/j.1574-6941.2010.00873.x 20455934

[44] Jones RT, Robeson MS, Lauber CL, Hamady M, Knight R, Fierer N. A comprehensive survey of soil acidobacterial diversity using pyrosequencing and clone library analyses. ISME J. 2009;3: 442–53. 10.1038/ismej.2008.127 19129864 PMC2997719

[45] Blasiak LC, Schmidt AW, Andriamiarinoro H, Mulaw T, Rasolomampianina R, Applequist WL, et al Bacterial communities in Malagasy soils with differing levels of disturbance affecting botanical diversity. PLoS One. 2014;9: e85097 10.1371/journal.pone.0085097 24465484 PMC3896373

[46] Lauber CL, Strickland MS, Bradford MA, Fierer N. The influence of soil properties on the structure of bacterial and fungal communities across land-use types. Soil Biol Biochem. 2008;40: 2407–2415.

[47] Li H, Smith SE, Holloway RE, Zhu Y, Smith FA. Arbuscular mycorrhizal fungi contribute to phosphorus uptake by wheat grown in a phosphorus-fixing soil even in the absence of positive growth responses. New Phytol. 2006;172: 536–43. 10.1111/j.1469-8137.2006.01846.x 17083683

[48] Schmidt SK, Wilson KL, Meyer AF, Gebauer MM, King AJ. Phylogeny and ecophysiology of opportunistic “snow molds” from a subalpine forest ecosystem. Microb Ecol. 2008;56: 681–7. 10.1007/s00248-008-9387-6 18443847

[49] Roesch LFW, Fulthorpe RR, Riva A, Casella G, Hadwin AKM, Kent AD, et al Pyrosequencing enumerates and contrasts soil microbial diversity. ISME J. 2007;1: 283–90. 10.1038/ismej.2007.53 18043639 PMC2970868

[50] Koleff P, Gaston KJ, Lennon JJ. Measuring beta diversity for presence-absence data. J Anim Ecol. 2003;72: 367–382.

