# Supplementary material for: Microbial Response to Soil Liming of Damaged Ecosystems Revealed by Pyrosequencing and Phospholipid Fatty Acid Analyses
Source: PLoS One. 2017 Jan 4;12(1):e0168497. doi: 10.1371/journal.pone.0168497 (PMC5215397; doi:10.1371/journal.pone.0168497)
Supplement: S1 Table — (DOCX) [file pone.0168497.s001.docx]

S1 Table: Bacterial species and their relative abundance identified from soil samples from the Greater Sudbury Region.

| **Bacterial species** | **Limed sites** | **Unlimed sites** |
| --- | --- | --- |
| 1. *Achromobacter* spp. | 18.75a (± 5.15) | 12.75a (± 4.32) |
| 1. *Acidicaldus* spp. | 30.00a (± 4.69) | 38.75a (± 4.87) |
| 1. *Acidimicrobiales* spp. | 13.75a (± 6.68) | 0.50b (± 0.58) |
| 1. *Acidimicrobium* spp. | 0.00a (± 0.00) | 2.75a (± 3.18) |
| 1. *Acidiphilium* spp. | 8.75a (± 8.99) | 4.50a (± 3.42) |
| 1. *Acidisphaera* spp. | 6.50a (± 2.45) | 11.50b (± 4.73) |
| 1. *Acidobacterium* spp. | 159.50a (± 10.74) | 683.75b (± 11.78) |
| 1. *Acidocella* spp. | 15.25a (± 5.70) | 5.00b (± 2.94) |
| 1. *Acidomonas baliensis* | 0.00a (± 0.00) | 1.00a (± 1.15) |
| 1. *Acidomonas methanolica* | 0.00a (± 0.00) | 1.50a (± 1.73) |
| 1. *Acidothermus* spp. | 4.75a (± 4.09) | 8.50a (± 2.89) |
| 1. *Acidovorax* spp. | 0.25a (± 0.29) | 3.25a (± 2.28) |
| 1. *Actinoplanes ferrugineus* | 3.25a (± 2.18) | 0.00a (± 0.00) |
| 1. *Afipia broomeae* | 423.25a (± 9.00) | 314.75b (± 13.03) |
| 1. *Afipia felis* | 12.75a (± 3.89) | 4.75b (± 2.37) |
| 1. *Afipia* spp. | 0.00a (± 0.00) | 2.00a (± 1.63) |
| 1. *Anaerolinea* spp. | 0.00a (± 0.00) | 7.75a (± 6.26) |
| 1. *Anderseniella* spp. | 2.00a (± 1.63) | 3.50a (± 2.73) |
| 1. *Aquicella* spp. | 49.75a (± 7.68) | 300.75b (± 18.48) |
| 1. *Arenimonas* spp. | 23.25a (± 4.88) | 1.25b (± 1.09) |
| 1. *Armatimonas* spp. | 0.25a (±0.29) | 0.00a (± 0.00) |
| 1. *Arthrobacter* spp. | 4.00a (± 4.62) | 0.00a (± 0.00) |
| 1. *Azospira* spp. | 20.75a (± 5.46) | 38.00b (± 4.51) |
| 1. *Azovibrio* spp. | 0.25a (± 0.29) | 0.00a (± 0.00) |
| 1. *Blastochloris* spp. | 1.00a (± 1.15) | 1.00a (± 0.82) |
| 1. *Blastochloris viridis* | 3.00a (± 3.46) | 0.00a (± 0.00) |
| 1. *Blastomonas* spp. | 0.00a (± 0.00) | 6.25b (± 2.23) |
| 1. *Bosea* spp. | 2.25a (± 2.23) | 0.00a (± 0.00) |
| 1. *Bradyrhizobium elkanii* | 2.75a (± 3.18) | 0.00a (± 0.00) |
| 1. *Bradyrhizobium* spp. | 69.75a (± 6.52) | 29.50b (± 5.81) |
| 1. *Burkholderia caryophylli* | 1.00a (± 0.47) | 0.25a (± 0.29) |
| 1. *Burkholderia* spp. | 2.25a (± 1.91) | 11.00b (± 2.23) |
| 1. *Byssovorax* spp. | 1.00a (± 1.15) | 0.00a (± 0.00) |
| 1. *Caldanaerobacter thermoanaerobacter* sp. | 0.00a (± 0.00) | 12.00a (± 13.86) |
| 1. *Caldanaerobacter thermoanaerobacter tengcongensis* | 5.50a (± 1.93) | 20.00b (± 4.36) |
| 1. *Caldilinea* spp. | 5.25 (± 6.06) | 5.50 (± 6.35) |
| 1. *Candidatus koribacter versatilis* | 0.25a (± 0.29) | 2.00a (± 1.63) |
| 1. *Candidates alysiosphaera* spp. | 20.50a (± 5.72) | 5.50b (± 2.77) |
| 1. *Candidates captivus* spp. | 1.00a (± 1.15) | 0.00a (± 0.00) |
| 1. *Candidates chloracidobacterium* spp. | 27.75a (± 7.93) | 20.25a (± 4.44) |
| 1. *Candidates chlorothrix* spp. | 0.00a (± 0.00) | 7.00a (± 8.08) |
| 1. *Candidates entotheonella* spp. | 70.75a (± 9.01) | 0.00b (± 0.00) |
| 1. *Candidates koribacter* spp. | 23.25a (± 9.68) | 262.75b (± 16.79) |
| 1. *Candidates microthrix* spp. | 1.75a (± 1.66) | 0.00a (± 0.00) |
| 1. *Candidates solibacter* spp. | 137.75a (± 12.37) | 286.5b (± 14.54) |
| 1. *Candidates xiphinematobacter* spp. | 5.00a (± 1.59) | 0.75b (± 0.87) |
| 1. *Caulobacter* spp. | 8.00a (± 3.74) | 24.75b (± 4.77) |
| 1. *Caulobacter vibrioides* | 4.25a (± 2.18) | 10.25a (± 8.94) |
| 1. *Collimonas fungivorans* | 0.00a (± 0.00) | 2.25a (± 2.60) |
| 1. *Colwellia* spp. | 7.50a (± 3.32) | 40.00b (± 4.34) |
| 1. *Comamonas* spp. | 0.00a (± 0.00) | 1.00a (± 1.15) |
| 1. *Conexibacter* spp. | 21.25a (± 7.61) | 51.00b (± 8.39) |
| 1. *Coprothermobacter* spp. | 0.75a (± 0.55) | 3.25a (± 1.60) |
| 1. *Corynebacteriales* spp. | 3.00a (± 3.46) | 0.00a (± 0.00) |
| 1. *Crossiella* spp. | 5.50a (± 3.84) | 7.00a (± 4.76) |
| 1. *Cryptosporangium japonicum* | 0.25a (± 0.29) | 0.00a (± 0.00) |
| 1. *Cupriavidus pinatubonensis* | 3.25a (± 3.75) | 0.00a (± 0.00) |
| 1. *Defluviicoccus* spp. | 25.50a (±10.34) | 14.25a (± 3.95) |
| 1. *Dermatophilus* spp. | 0.00a (± 0.00) | 2.75a (± 3.18) |
| 1. *Dongia* spp. | 5.00a (± 2.40) | 12.75a (± 10.81) |
| 1. *Dyella* spp. | 6.00a (± 6.93) | 0.00a (± 0.00) |
| 1. *Edaphobacter modestum* | 7.25a (± 2.80) | 11.75a (± 3.95) |
| 1. *Edaphobacter* spp. | 1.00a (± 1.15) | 10.50b (± 2.74) |
| 1. *Escherichia shigella* spp. | 1.75a (± 1.19) | 5.50a (± 5.97) |
| 1. *Ferruginibacter* spp. | 8.00a (± 1.25) | 18.00b (± 2.38) |
| 1. *Filomicrobium* spp. | 2.50a (± 2.89) | 65.75b (± 7.39) |
| 1. *Fluviicola* spp. | 0.75a (± 0.87) | 0.00a (± 0.00) |
| 1. *Frankia* spp. | 3.50a (± 1.45) | 2.75a (± 1.44) |
| 1. *Geitlerinema* spp. | 0.00a (± 0.00) | 1.25a (± 1.44) |
| 1. *Gemmata* spp. | 11.75a (± 3.24) | 11.50a (± 2.37) |
| 1. *Gemmatimonas* spp. | 8.00a (± 1.74) | 18.75b (± 2.40) |
| 1. *Geobacillus* spp. | 41.25a (± 3.81) | 167.75b (± 13.03) |
| 1. *Granulicella* spp. | 74.50a (± 10.48) | 186.75b (± 16.68) |
| 1. *Haliangium* spp. | 2.75a (± 2.47) | 0.50a (± 0.58) |
| 1. *Hirschia* spp. | 19.75a (± 3.67) | 21.50a (± 2.81) |
| 1. *Hymenobacter* spp. | 0.00a (± 0.00) | 10.75a (± 12.41) |
| 1. *Hyphomicrobium* spp. | 1.50a (± 1.11) | 2.75a (± 3.18) |
| 1. *Iamia* spp. | 0.00a (± 0.00) | 5.75b (± 1.98) |
| 1. *Inquilinus* spp. | 2.00a (± 0.82) | 3.50a (± 3.32) |
| 1. *Kineosporia* spp. | 6.00a (± 2.79) | 0.00b (± 0.00) |
| 1. *Kribbella* spp. | 1.25a (± 1.44) | 0.00a (± 0.00) |
| 1. *Ktedonobacter* spp. | 12.00a (± 3.47) | 3.25b (± 2.28) |
| 1. *Labrys* spp. | 28.00a (± 4.65) | 14.75b (± 3.91) |
| 1. *Leptothrix* spp. | 1.00a (± 1.15) | 2.75a (± 1.85) |
| 1. *Luteibacter* spp. | 1.00a (± 1.15) | 0.00a (± 0.00) |
| 1. *Magnetospirillum* sp. | 1.75a (± 2.02) | 0.00a (± 0.00) |
| 1. *Marmoricola* spp. | 16.25a (± 15.16) | 0.00a (± 0.00) |
| 1. *Massilia* spp. | 2.00a (± 1.25) | 0.00a (± 0.00) |
| 1. *Massilia timonae* | 2.75a (± 3.18) | 0.00a (± 0.00) |
| 1. *Methylocystis* spp. | 8.50a (± 2.11) | 7.75a (± 1.38) |
| 1. *Methylosinus sporium* | 0.50a (± 0.58) | 0.00a (± 0.00) |
| 1. *Methylosinus* spp. | 0.75a (± 0.87) | 0.00a (± 0.00) |
| 1. *Methylosinus trichosporium* | 0.00a (± 0.00) | 6.25b (± 2.84) |
| 1. *Methylotenera* spp. | 6.75a (± 7.79) | 0.00a (± 0.00) |
| 1. *Methyloversatilis* spp. | 3.25a (± 1.44) | 0.00b (± 0.00) |
| 1. *Metyhlovirgula* spp. | 2.00a (± 1.41) | 2.00a (± 1.94) |
| 1. *Mitochondria marchantia polymorpha* | 0.00a (± 0.00) | 2.75a (± 3.18) |
| 1. *Mucilaginibacter* spp. | 0.00a (± 0.00) | 3.25a (± 3.75) |
| 1. *Mycobacterium celatum* | 1.00a (± 0.82) | 0.00a (± 0.00) |
| 1. *Mycobacterium insubricum* | 0.00a (± 0.00) | 0.25a (± 0.29) |
| 1. *Mycobacterium riyadhense* | 2.25a (± 2.60) | 19.75b (± 6.88) |
| 1. *Mycobacterium* spp. | 0.00a (± 0.00) | 1.75a (± 1.36) |
| 1. *Nitrosococcus* spp. | 56.00a (± 7.68) | 81.50b (± 4.20) |
| 1. *Nitrospira freshwater sediment* | 0.75a (± 0.87) | 0.00a (± 0.00) |
| 1. *Nocardioides* spp. | 4.25a (± 4.91) | 0.00a (± 0.00) |
| 1. *Novosphingobium* spp. | 2.50a (± 2.19) | 0.00a (± 0.00) |
| 1. *Opitutus* spp. | 2.25a (± 2.60) | 0.25a (± 0.29) |
| 1. *Parasegetibacter* spp. | 1.00a (± 1.15) | 0.00a (± 0.00) |
| 1. *Parvularcula* spp. | 0.00a (± 0.00) | 1.25a (± 1.44) |
| 1. *Pedomicrobium* spp. | 16.00a (± 2.88) | 4.50b (± 3.12) |
| 1. *Phenylobacterium* spp. | 17.50a (± 5.67) | 3.00b (± 2.45) |
| 1. *Pilimelia* spp. | 58.00a (± 3.32) | 1.50b (± 1.37) |
| 1. *Pirellula* spp. | 3.00a (± 0.05) | 0.00b (± 0.00) |
| 1. *Planctomyces* spp. | 0.25a (± 0.29) | 10.00b (**±** 2.60) |
| 1. *Pleomorphomonas oryzae* | 0.25a (± 0.29) | 0.25a (± 0.29) |
| 1. *Polaromonas* spp. | 0.00a (± 0.00) | 5.50a (± 6.35) |
| 1. *Pseudolabrys* spp. | 43.25a (± 7.49) | 3.50b (± 0.75) |
| 1. *Rhizobium* spp. | 2.75a (± 3.18) | 3.00a (± 1.25) |
| 1. *Rhodobacter* spp. | 8.00a (± 1.58) | 0.00b (± 0.00) |
| 1. *Rhodobium orientis* | 0.00a (± 0.00) | 1.25a (± 1.44) |
| 1. *Rhodopirellula* spp. | 1.00a (± 1.15) | 6.00b (± 1.16) |
| 1. *Rhodoplanes elegans* | 3.75a (± 4.33) | 0.00a (± 0.00) |
| 1. *Rhodoplanes* spp. | 115.75a (± 5.19) | 120.5a (± 7.93) |
| 1. *Rhodovastum atsumiense* | 0.50a (± 0.58) | 1.00a (± 0.82) |
| 1. *Rhodovibrio* spp. | 1.00a (± 0.82) | 8.50a (± 8.35) |
| 1. *Roseomonas ruber* | 0.75a (± 0.55) | 1.50a (± 1.76) |
| 1. *Sciscionella* spp. | 1.00a (± 1.15) | 44.25b (± 4.25) |
| 1. *Sideroxydans* spp. | 0.25a (± 0.29) | 0.00a (± 0.00) |
| 1. *Simplicispira* spp. | 0.50a (± 0.33) | 3.50a (± 4.04) |
| 1. *Singulisphaera nostocoida limicola* | 0.00a (± 0.00) | 4.50a (± 5.20) |
| 1. *Skermanella* spp. | 19.75a (± 10.84) | 105.75b (± 25.88) |
| 1. *Sneathiella* spp. | 2.25a (± 0.66) | 0.00b (± 0.00) |
| 1. *Solirubrobacter* spp. | 23.75a (± 3.02) | 15.00b (± 5.94) |
| 1. *Sorangiineae* spp. | 2.75a (± 3.18) | 1.00a (± 1.15) |
| 1. *Sorangium* spp. | 2.00a (± 1.05) | 21.00b (± 6.82) |
| 1. *Sphingomonas caulobacter leidyia* | 1.50a (± 1.73) | 0.00a (± 0.00) |
| 1. *Sphingomonas* spp. | 0.50a (± 0.58) | 0.00a (± 0.00) |
| 1. *Streptomyces lincolnensis* | 0.25a (± 0.29) | 0.00a (± 0.00) |
| 1. *Telmatospirillum* spp. | 0.25a (± 0.29) | 2.50a (± 2.52) |
| 1. *Tepidamorphus* spp. | 1.25a (± 1.44) | 0.00a (± 0.00) |
| 1. *Thermogemmatispora foliorum* | 0.00a (± 0.00) | 4.75a (± 5.48) |
| 1. *Thermogemmatispora onikobensis* | 0.00a (± 0.00) | 9.00a (± 10.39) |
| 1. *Thermomonas* spp. | 0.25a (± 0.29) | 7.75b (± 2.84) |
| 1. *Thermosporothrix* spp. | 12.75a (± 4.84) | 343.75b (± 21.30) |
| 1. *Thioalkalispira* spp. | 8.50a (± 5.11) | 134.25b (± 10.00) |
| 1. *Tistlia* spp. | 0.00a (± 0.00) | 1.25a (± 1.44) |
| 1. *TM7* | 7.75a (± 3.07) | 24.50b (± 4.12) |
| 1. *Victivallis* spp. | 0.00a (± 0.00) | 1.00b (± 0.00) |
| 1. *Zavarzinella* spp. | 0.00a (± 0.00) | 2.50a (± 2.19) |

Results are expressed as mean values ± standard error

Means in rows with a common letter are not significantly different based on T-test (p ≥ 0.05).

Limed and Unlimed sites: Daisy Lake 2 (site 1), Wahnapitae Hydro-Dam (site 2), Kelly Lake (site 3), and Kingsway (site 4).
