# Supplementary material for: Microbial Response to Soil Liming of Damaged Ecosystems Revealed by Pyrosequencing and Phospholipid Fatty Acid Analyses
Source: PLoS One. 2017 Jan 4;12(1):e0168497. doi: 10.1371/journal.pone.0168497 (PMC5215397; doi:10.1371/journal.pone.0168497)
Supplement: S2 Table — (DOCX) [file pone.0168497.s002.docx]

S2 Table: Fungal species and their relative abundance identified from soil samples from the Greater Sudbury Region.

| **Fungi species** | **Limed sites** | **Unlimed sites** |
| --- | --- | --- |
| 1. *Agaricomycotina sp.* | 109.25a (± 39.71) | 306.50b (±18.61) |
| 1. *Amanita muscaria* | 70.75a (± 11.70) | 4.00b (± 4.62) |
| 1. *Amanita sp.* | 1.00a (± 0.82) | 0.00a (± 0.00) |
| 1. *Archaeorhizomyces finlayi* | 16.75a (± 19.34) | 0.00a (± 0.00) |
| 1. *Ascomycete sp.* | 0.00a (± 0.00) | 18.50b (± 7.57) |
| 1. *Ascomycota sp.* | 20.00a (± 19.74) | 199.00b (± 19.28) |
| 1. *Atheliaceae sp.* | 0.50a (± 0.58) | 0.00a (± 0.00) |
| 1. *Basidiomycota sp.* | 1.50a (± 1.37) | 127.75b (± 14.51) |
| 1. *Calicium salicinum* | 2.25a (± 2.60) | 21.00b (± 4.25) |
| 1. *Cenococcum geophilum* | 42.75a (± 4.84) | 0.00b (± 0.00) |
| 1. *Chaetomella oblonga* | 2.75a (± 3.18) | 1.00a (± 1.15) |
| 1. *Cladonia coniocraea* | 0.00a (± 0.00) | 9.50a (± 10.97) |
| 1. *Clavulinaceae sp.* | 42.75a (± 49.36) | 0.00a (± 0.00) |
| 1. *Cortinariaceae sp.* | 10.00a (± 11.55) | 0.00a (± 0.00) |
| 1. *Cortinarius flos paludis* | 636.00a (± 34.39) | 0.00b (± 0.00) |
| 1. *Cryptococcus podzolicus* | 87.50a (± 8.34) | 25.75b (± 15.86) |
| 1. *Dermateaceae sp.* | 11.75a (± 7.52) | 52.25b (± 4.18) |
| 1. *Dibaeis baeomyces* | 0.00a (± 0.00) | 798.50b (± 22.03) |
| 1. *Dothideomycetes sp.* | 5.00a (± 2.54) | 33.75a (± 24.68) |
| 1. *Elaphomyces muricatus* | 0.00a (± 0.00) | 0.75a (± 0.55) |
| 1. *Ericoid mycorrhizal sp.* | 41.00a (± 4.49) | 0.00b (± 0.00) |
| 1. *Fungi fungal sp.* | 5.75a (± 3.84) | 16.25b (± 4.16) |
| 1. *Fungi mycorrhizal fungal sp.* | 2.25a (± 1.91) | 10.50a (± 12.12) |
| 1. *Fusarium oxysporum* | 7.75a (± 8.20) | 0.00a (± 0.00) |
| 1. *Gyoerffyella sp.* | 1.75a (± 1.66) | 0.75a (± 0.55) |
| 1. *Helotiaceae sp.* | 65.00a (± 4.53) | 96.75b (± 9.61) |
| 1. *Helotiales sp.* | 31.50a (± 5.36) | 53.25b (± 7.63) |
| 1. *Herpotrichiellaceae sp.* | 3.50a (± 2.03) | 23.75b (± 12.44) |
| 1. *Inocybe abjecta* | 78.00a (± 13.53) | 0.00b (± 0.00) |
| 1. *Inocybe fuscidula* | 351.75a (± 40.17) | 0.00b (± 0.00) |
| 1. *Inocybe lacera* | 0.25a (± 0.29) | 0.00a (± 0.00) |
| 1. *Laccaria proxima* | 46.50a (± 16.01) | 197.75b (± 17.12) |
| 1. *Laccaria sp.* | 12.50a (± 12.94) | 15.75a (± 18.19) |
| 1. *Lactarius camphoratus* | 0.00a (± 0.00) | 4.25a (± 4.91) |
| 1. *Lactarius fuscus* | 12.75a (± 12.52) | 2.75a (± 2.47) |
| 1. *Leotia viscosa* | 1.00a (± 1.15) | 36.25b (± 4.86) |
| 1. *Leotiomycetes sp.* | 1.00a (± 1.15) | 23.50b (± 5.62) |
| 1. *Magnaporthales sp.* | 1.25a (± 1.44) | 1.25a (± 1.44) |
| 1. *Mortierella sp.* | 2.50a (± 2.89) | 14.00a (± 14.31) |
| 1. *Mortierellales sp.* | 8.00a (± 6.99) | 59.25b (± 6.25) |
| 1. *Mucoromycotina sp.* | 0.00a (± 0.00) | 28.75a (± 33.20) |
| 1. *Myrothecium cinctum* | 18.50a (± 21.36) | 0.00a (± 0.00) |
| 1. *Myxotrichaceae sp.* | 22.00a (± 16.46) | 21.00a (± 24.25) |
| 1. *Oidiodendron maius* | 9.00a (± 4.03) | 26.50b (± 11.27) |
| 1. *Penicillium montanense* | 41.25a (± 4.63) | 16.00b (± 5.08) |
| 1. *Pezizomycotina sp.* | 140.25a (± 10.70) | 67.50b (± 15.66) |
| 1. *Phialocephala fortinii* | 9.25a (± 5.24) | 15.50a (± 11.21) |
| 1. *Piloderma lanatum* | 0.00a (± 0.00) | 9.25a (± 10.68) |
| 1. *Pyronemataceae sp.* | 0.00a (± 0.00) | 24.75a (± 28.58) |
| 1. *Russula aeruginea* | 12.00a (± 9.80) | 31.75a (± 35.13) |
| 1. *Russula gracilis* | 123.50a (± 20.70) | 0.25b (± 0.29) |
| 1. *Russula sp.* | 170.75a (± 18.92 ) | 454.25b (± 24.52) |
| 1. *Russula sphagnophila* | 44.50a (± 4.28) | 86.75b (± 9.11) |
| 1. *Russula ventricosipes* | 0.25a (± 0.29) | 5.00a (± 5.77) |
| 1. *Russula vesca* | 41.00a (± 47.34) | 24.50a (± 28.29) |
| 1. *Russulaceae sp.* | 359.25a (± 414.83) | 0.00a (± 0.00) |
| 1. *Scleroderma citrinum* | 0.00a (± 0.00) | 80.50a (± 58.61) |
| 1. *Sebacinaceae sp.* | 14.25a (± 16.45) | 159.50b (± 22.06) |
| 1. *Sordariales sp.* | 1.00a (± 0.82) | 0.00a (± 0.00) |
| 1. *Sordariomycetes sp.* | 0.25a (± 0.29) | 0.25a (± 0.29) |
| 1. *Suillus brevipes* | 206.50a (± 20.04) | 0.00b (± 0.00) |
| 1. *Thelephoraceae sp.* | 1555.00a (± 277.48) | 0.25b (± 0.29) |
| 1. *Tomentella sp.* | 1.50a (± 0.58) | 8.00a (± 9.24) |
| 1. *Tremella diploschistina* | 0.00a (± 0.00) | 3.75a (± 4.33) |
| 1. *Tricholoma ustale* | 30.50a (± 34.45) | 0.00a (± 0.00) |
| 1. *Tricholomataceae sp.* | 36.75a (± 42.44) | 0.00a (± 0.00) |
| 1. *Tylospora asterophora* | 0.00a (± 0.00) | 0.50a (± 0.58) |
| 1. *Venturiales sp.* | 3.00a (± 2.05) | 2.75a (± 3.18) |
| 1. *Wilcoxina mikolae* | 53.75a (± 37.51) | 0.00a (± 0.00) |
| 1. *Xerocomus badius* | 0.25a (± 0.29) | 9.25a (± 10.68) |

Results are expressed as mean values ± standard error

Means in rows with a common letter are not significantly different based on T-test (p ≥ 0.05).

Limed and Unlimed sites: Daisy Lake 2 (site 1), Wahnapitae Hydro-Dam (site 2), Kelly Lake (site 3), and Kingsway (site 4).
