# Supplementary material for: Microbial Response to Soil Liming of Damaged Ecosystems Revealed by Pyrosequencing and Phospholipid Fatty Acid Analyses
Source: PLoS One. 2017 Jan 4;12(1):e0168497. doi: 10.1371/journal.pone.0168497 (PMC5215397; doi:10.1371/journal.pone.0168497)
Supplement: S3 Table — (DOCX) [file pone.0168497.s003.docx]

S3 Table: Site-specific bacterial species from the Greater Sudbury Region (number in parentheses represent relative abundance).

|  | **Limed sites** | **Unlimed** |
| --- | --- | --- |
| 1 | *Actinoplanes ferrugineus* (3.25) | *Acidomonas baliensis* (1.00) |
| 2 | *Arthrobacter* spp. (4.00) | *Acidomonas methanolica* (1.50) |
| 3 | *Bradyrhizobium elkanii* (2.75) | *Afipia* spp. (2.00) |
| 4 | *Byssovorax* spp. (1.00) | *Anaerolinea* spp. (7.75) |
| 5 | *Candidates captivus* spp. (1.00) | *Blastomonas* spp. (6.25) |
| 6 | *Candidates microthrix* spp. (1.75) | *Caldanaerobacter thermoanaerobacter* sp. (12.00) |
| 7 | *Cryptosporangium japonicum* (0.25) | *Candidates chlorothrix* spp. (7.00) |
| 8 | *Kribbella* spp. (1.25) | *Comamonas* spp. (1.00) |
| 9 | *Luteibacter* spp. (1.00) | *Dermatophilus* spp. (2.75) |
| 10 | *Methylosinus* spp. (0.75) | *Geitlerinema* spp. (1.25) |
| 11 | *Methylotenera* spp. (6.75) | *Mitochondria marchantia polymorpha* (2.75) |
| 12 | *Methyloversatilis* spp. (3.25) | *Mucilaginibacter* spp. (3.25) |
| 13 | *Nitrospira freshwater sediment* (0.75) | *Mycobacterium insubricum* (0.25) |
| 14 | *Nocardioides* spp. (4.25) | *Mycobacterium* spp. (1.75) |
| 15 | *Parasegetibacter* spp. (1.00) | *Parvularcula* spp.(1.25) |
| 16 | *Pirellula* spp. (3.00) | *Polaromonas* spp. (5.50) |
| 17 | *Rhodobacter* spp. (8.00) | *Rhodobium orientis* (1.25) |
| 18 | *Rhodoplanes elegans* (3.75) | *Tistlia* spp. (1.25) |
| 19 | *Sphingomonas caulobacter leidyia* (1.5) | *Thermogemmatispora foliorum* (4.75) |
| 20 | *Streptomyces lincolnensis* (0.25) | *Thermogemmatispora onikobensis* (9.00) |
| 21 |  | *Victivallis* spp. (1.00) |

Limed and Unlimed sites: Daisy Lake 2 (site 1), Wahnapitae Hydro-Dam (site 2), Kelly Lake (site 3), and Kingsway (site 4).
