# Supplementary material for: Microbial Response to Soil Liming of Damaged Ecosystems Revealed by Pyrosequencing and Phospholipid Fatty Acid Analyses
Source: PLoS One. 2017 Jan 4;12(1):e0168497. doi: 10.1371/journal.pone.0168497 (PMC5215397; doi:10.1371/journal.pone.0168497)
Supplement: S4 Table — (DOCX) [file pone.0168497.s004.docx]

S4 Table: Weighed UniFrac distance matrix between sites for bacterial communities.

|  | **Daisy Lake 2 Limed** | **Daisy Lake 2 Unlimed** | **Wahnapitae Hydro-Dam Limed** | **Wahnapitae Hydro-Dam Unlimed** | **Kelly Lake Limed** | **Kelly Lake Unlimed** | **Kingsway Limed** | **Kingsway Unlimed** |
| --- | --- | --- | --- | --- | --- | --- | --- | --- |
| Daisy Lake 2 Limed | 0.00 | 0.31 | 0.24 | 0.30 | 0.16 | 0.23 | 0.21 | 0.20 |
| Daisy Lake 2 Unlimed |  | 0.00 | 0.35 | 0.47 | 0.37 | 0.30 | 0.36 | 0.41 |
| Wahnapitae Hydro-Dam Limed |  |  | 0.00 | 0.40 | 0.24 | 0.25 | 0.18 | 0.32 |
| Wahnapitae Hydro-Dam Unlimed |  |  |  | 0.00 | 0.30 | 0.36 | 0.34 | 0.19 |
| Kelly Lake Limed |  |  |  |  | 0.00 | 0.21 | 0.17 | 0.21 |
| Kelly Lake Unlimed |  |  |  |  |  | 0.00 | 0.21 | 0.29 |
| Kingsway Limed |  |  |  |  |  |  | 0.00 | 0.26 |
| Kingsway Unlimed |  |  |  |  |  |  |  | 0.00 |
