# Supplementary material for: Microbial Response to Soil Liming of Damaged Ecosystems Revealed by Pyrosequencing and Phospholipid Fatty Acid Analyses
Source: PLoS One. 2017 Jan 4;12(1):e0168497. doi: 10.1371/journal.pone.0168497 (PMC5215397; doi:10.1371/journal.pone.0168497)
Supplement: S5 Table — (DOCX) [file pone.0168497.s005.docx]

S5 Table: Fungi species and its relative abundance identified unique to sites from the GSR.

|  | **Limed sites** | **Unlimed sites** |
| --- | --- | --- |
| 1 | *Archaeorhizomyces finlayi* (16.75) | *Ascomycete* sp*.* (18.50) |
| 2 | *Cortinarius flos paludis* (636.00) | *Cladonia coniocraea* (9.50) |
| 3 | *Fusarium oxysporum* (7.75) | *Dibaeis baeomyces* (798.50) |
| 4 | *Inocybe abject* (78.00) | *Piloderma lanatum* (9.25) |
| 5 | *Inocybe fuscidula* (351.75) | *Tremella diploschistina* (3.75) |
| 6 | *Myrothecium cinctum* (18.50) |  |
| 7 | *Suillus brevipes* (206.50) |  |
| 8 | *Tricholomataceae* sp. (36.75) |  |
| 9 | *Tricholoma ustale* (30.50) |  |
| 10 | *Wilcoxina mikolae* (53.75) |  |

Limed and Unlimed sites: Daisy Lake 2 (site 1), Wahnapitae Hydro-Dam (site 2), Kelly Lake (site 3), and Kingsway (site 4).
