# Supplementary material for: Microbial Response to Soil Liming of Damaged Ecosystems Revealed by Pyrosequencing and Phospholipid Fatty Acid Analyses
Source: PLoS One. 2017 Jan 4;12(1):e0168497. doi: 10.1371/journal.pone.0168497 (PMC5215397; doi:10.1371/journal.pone.0168497)
Supplement: S6 Table — (DOCX) [file pone.0168497.s006.docx]

S6 Table: Weighed UniFrac distance matrix between sites for fungal community.

|  | **Daisy Lake 2 Limed** | **Daisy Lake 2 Unlimed** | **Wahnapitae Hydro-Dam Limed** | **Wahnapitae Hydro-Dam Unlimed** | **Kelly Lake Limed** | **Kelly Lake Unlimed** | **Kingsway Limed** | **Kingsway Unlimed** |
| --- | --- | --- | --- | --- | --- | --- | --- | --- |
| Daisy Lake 2 Limed | 0.00 | 0.95 | 0.99 | 0.97 | 0.89 | 0.94 | 0.97 | 0.95 |
| Daisy Lake 2 Unlimed |  | 0.00 | 1.00 | 0.98 | 0.97 | 0.91 | 0.99 | 0.96 |
| Wahnapitae Hydro-Dam Limed |  |  | 0.00 | 1.00 | 0.99 | 1.00 | 0.88 | 1.00 |
| Wahnapitae Hydro-Dam Unlimed |  |  |  | 0.00 | 0.94 | 0.98 | 0.98 | 0.97 |
| Kelly Lake Limed |  |  |  |  | 0.00 | 0.90 | 0.95 | 0.91 |
| Kelly Lake Unlimed |  |  |  |  |  | 0.00 | 0.97 | 0.93 |
| Kingsway Limed |  |  |  |  |  |  | 0.00 | 0.75 |
| Kingsway Unlimed |  |  |  |  |  |  |  | 0.00 |
